# Clinical Performance of the Xpert^®^ CT/NG Test for Detection of *Chlamydia trachomatis* and *Neisseria gonorrhoeae*: A Multicenter Evaluation in Chinese Urban Hospitals

**DOI:** 10.3389/fcimb.2021.784610

**Published:** 2022-01-03

**Authors:** Yan Han, Mei-Qin Shi, Qing-Ping Jiang, Wen-Jing Le, Xiao-Lin Qin, Han-Zhen Xiong, He-Ping Zheng, Fred C. Tenover, Yi-Wei Tang, Yue-Ping Yin

**Affiliations:** ^1^ Institute of Dermatology, Chinese Academy of Medical Sciences & Peking Union Medical College, Nanjing, China; ^2^ National Center for STD Control, Chinese Center for Disease Control and Prevention, Nanjing, China; ^3^ Depatment of Pathology, Third Affiliated Hospital, Guangzhou Medical University, Guangzhou, China; ^4^ Dermatology Hospital, Southern Medical University, Guangzhou, China; ^5^ Department of Medical and Scientific Affairs, Cepheid, Sunnyvale, CA, United States; ^6^ Department of Medical and Scientific Affairs, Danaher Diagnostic Platform China/Cepheid, Shanghai, China

**Keywords:** *Chlamydia trachomatis*, *Neisseria gonorrhoeae*, molecular testing, urine, cervical swabs, point-of-care testing

## Abstract

**Background:**

We aimed to evaluate the clinical performance of the GeneXpert^®^ (Xpert) CT/NG assay for the detection of *Chlamydia trachomatis* (CT) and *Neisseria gonorrhoeae* (NG) using urine and cervical swabs collected from patients in China.

**Methods:**

This study was conducted from September 2016 to September 2018 in three Chinese urban hospitals. The results from the Xpert CT/NG test were compared to those from the Roche cobas^®^ 4800 CT/NG test. Discordant results were confirmed by DNA sequence analysis.

**Results:**

In this study, 619 first void urine (FVU) specimens and 1,042 cervical swab specimens were included in the final dataset. There were no statistical differences between the results of the two tests for the detection of CT/NG in urine samples (*p* > 0.05), while a statistical difference was found in cervical swabs (*p* < 0.05). For CT detection, the sensitivity and specificity of the Xpert test were 100.0% (95%CI = 96.8–99.9) and 98.3% (95%CI = 96.6–99.2) for urine samples and 99.4% (95%CI = 96.5–100.0) and 98.6% (95%CI 97.5–99.2) for cervical swabs, respectively. For NG detection, the sensitivity and specificity of the Xpert test were 99.2% (95%CI = 94.9–100.0) and 100.0% (95%CI = 99.0–100.0) for urine and 100% (95%CI = 92.8–100.0) and 99.7% (95%CI = 99.0–99.9) for cervical swabs, respectively.

**Conclusion:**

The Xpert CT/NG test exhibited high sensitivity and specificity in the detection of CT and NG in both urine and cervical samples when compared to the reference results. The 90-min turnaround time for CT and NG detection at the point of care using Xpert may enable patients to receive treatment promptly.

## Introduction

*Chlamydia trachomatis* (CT) and *Neisseria gonorrhoeae* (NG) are two of the most common sexually transmitted bacterial pathogens across the world and are the main contributors to sexually transmitted infections (STIs) in China. Both infections can have serious sequelae, especially in women, including pelvic inflammatory disease, which can lead to ectopic pregnancy and infertility (Baud et al., 2008; Ginocchio et al., 2012) and facilitate the risk of HIV transmission (Cohen et al., 1999; Bernstein et al., 2010). The World Health Organization (WHO) estimated that the incident cases for CT were 127.2 million and for NG were 86.9 million in 2016 (Rowley et al., 2019). The average duration of CT is 1.4 years (Price et al., 2013), and that of NG is about 6 months in the absence of antimicrobial treatment (Grad et al., 2016). Most CT or NG infections are asymptomatic. The slower the clearance occurs, the higher the prevalence of untreated infection and the more effective a screening intervention is needed to be.

Traditionally, culture was used as the gold standard for the diagnosis of CT and NG. With the development of science and technology, nucleic acid amplification tests (NAATs) are currently recommended as the diagnostic methods for CT and NG in most high-income countries due to their high specificity and sensitivity (Garrett et al., 2016). The traditional culture method for CT is time-consuming, tedious, and typically requires cell culture preparations, such as McCoy, Hela 229, or Buffalo green monkey kidney cells. The samples need to be centrifuged, incubated for 48–72 h, and examined by microscopy (Barnes, 1989; Centers for Disease Control and Prevention, 2014). In contrast, nucleic acid amplification tests amplify the unique target sequences in a microorganism in real-time rapidly, which means identifying microorganisms directly in clinical specimens. However, this can potentially also be a long process. For example, most of the commercially available NAATs for CT and NG registered in China with the National Medical Products Administration (NMPA) require multiple steps and expensive equipment and take 1 or 2 days to generate results. On the other hand, the Cepheid GeneXpert^®^ (Xpert) CT/NG assay is a rapid NAAT assay that can be performed on the GeneXpert instrument platform in laboratories and is simple to operate. The Xpert test detects the DNA of CT and NG in specimens by nucleic acid amplification in approximately 90 min. Anywhere from 1 to 80 specimens can be processed simultaneously depending on the type of the instrument used and the number of samples processed per day. The easy-to-use modular cartridges minimize the processing steps and control contamination. The assay was approved by the United States Food and Drug Administration and was CE-IVD (European CE Marking for *In Vitro* Diagnostic devices) marked in 2012. In order to introduce this assay into China, we undertook this study to compare the Xpert CT/NG test to the Roche Cobas 4800CT/NG, which was approved by the China NMPA in 2014.

## Materials and Methods

### Study Population

This evaluation was conducted from September 2016 to September 2018 in three sites: 1) Institute of Dermatology, Chinese Academy of Medical Sciences and Peking Union Medical College, 2) Dermatology Hospital of Southern Medical University, and 3) The Third Affiliated Hospital of Guangzhou Medical University. All sites obtained institutional review board approval for the clinical study in accordance with the Declaration of Helsinki. The approval numbers for the study in three sites were 2016-LS-009, GDDHLS-20171201, and YLSJ-2018-002 for the Institute of Dermatology, the Chinese Academy of Medical Sciences and Peking Union Medical College, Dermatology Hospital of Southern Medical University, and The Third Affiliated Hospital of Guangzhou Medical University, respectively. The patients were ≥18 years of age and sought healthcare in sexually transmitted disease clinics in two dermatology hospitals or the Obstetrics and Gynecology Clinic in the general hospital. They signed an informed consent form and confirmed their willingness to provide urine or cervical swabs for this study.

### Specimen Collection

Female patients were encouraged to provide cervical swabs and first void urine (FVU), while male patients were asked to provide FVU. The FVU specimens were collected in a urine cup by the patients. Approximately 5–7 ml of urine was aliquoted into the urine collection device of each manufacturer, which contained a preservative. The remaining urine was transferred into a cryogenic vial and frozen at −20°C at the local hospitals. The healthcare technicians collected two cervical swabs, and the order of cervical specimen collection was randomized for transfer into the cobas^®^ or Cepheid^®^ PCR Media tube, such that swabs for each of the two test assays had equal opportunity to be collected first or second. All swab samples were collected and transported according to the package insert directions of each manufacturer. Leftover specimens were stored at 4°C refrigerators in the local hospitals.

### Laboratory Testing

The Xpert test was performed according to the instructions of the manufacturer instructions at each study site. Of the prepared sample, 300 μl was transferred into the sample chamber in the cartridge, and an elution reagent was pipetted into the cartridge. Then, the cartridge was inserted into the GeneXpert platform and the test was initiated. The test included a specimen adequacy control and an amplification control. The adequacy control is to ensure that there is sufficient human DNA in the sample, and the amplification control is to guarantee an effective amplification of nucleic acid in the specimen. Results included a specimen adequacy control result and an amplification control result. The results were reported in <2 h as positive or negative for chlamydia, positive or negative for gonorrhea, or indeterminate (reading invalid, error, or no result). When a test failed or the test was read as indeterminate, the specimen was retested one additional time using a new aliquot of the specimen, if available, and a new Xpert cartridge.

The cobas^®^ 4800 CT/NG assay (Roche Molecular Systems, Branchburg, NJ, USA) was used as the reference test to detect CT and NG according to the manufacturer’s instructions. The cobas^®^ x 480 instrument was used to extract nucleic acid from the urine and cervical samples and distribute it into the PCR reaction mixture. The cobas^®^ z 480 analyzer was used to fully automate PCR amplification and detection. The test results were automatically reported according to the preset computer algorithm. The results were reported as positive, negative, or invalid for CT; positive, negative, or invalid for NG; or failed. Repeated equivocal results were reported as invalid or failed.

### Data Analysis

Samples yielding indeterminate results in the second run after failing the first run were not included in the final dataset (Bristow et al., 2017). Concordance, positive percent agreement, and negative percent agreement were calculated for CT and NG for the urine and cervical swab samples. McNemar’s test was used to compare the performances of the Xpert and the Roche cobas CT/NG tests. Concordant results for specimens were defined as positive or negative if the Xpert and Roche cobas CT/NG assays gave the same results for CT or NG. For discordant results of CT or NG, the samples underwent DNA sequence analysis for CT or NG conducted by independent clinical laboratories in China (the Guangzhou and Nanjing central laboratories operated by KingMed Diagnostics). The sequencing results were used for discrepant analysis. The sensitivity and specificity of the Xpert assay for CT and NG were calculated by comparing with the final results for urine and cervical swabs, respectively.

## Results

**Figure 1:**
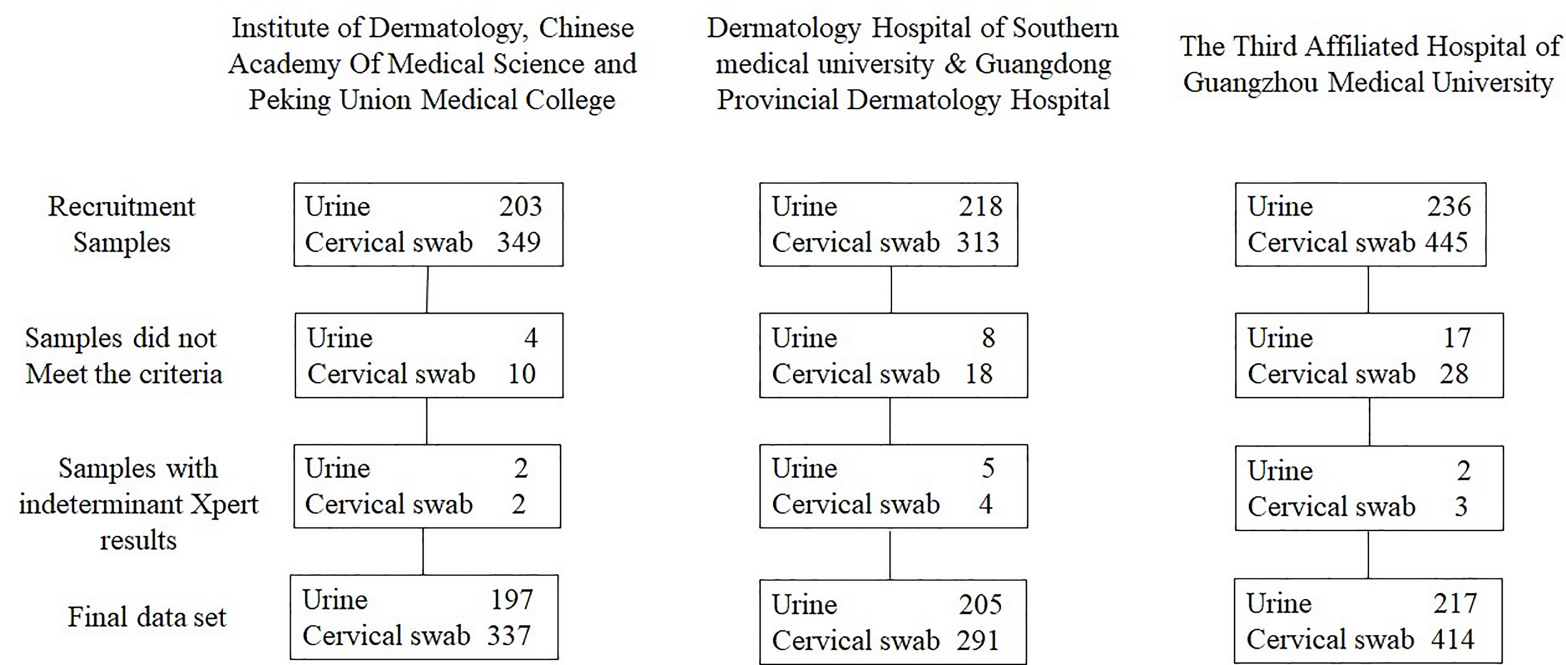
The flowchart for the sample collection.

During the study period, 657 participants provided FVU and 1,107 participants provided cervical swabs; 38 FVU specimens and 65 cervical swab specimens did not meet the study criteria or gave indeterminate Xpert results and were excluded from the analysis. Ultimately, 619 FVU specimens and 1,042 cervical swab specimens were included in the final dataset (**Figure 1**). The average age of the participants who provided FVU was 34.0 years (SD ± 9.95 years, range = 18–68 years), and 47.7% (295/619) were women. Furthermore, 197 (31.8%) urine specimens were obtained from the Institute of Dermatology, Chinese Academy of Medical Sciences, and Peking Union Medical College. The average age of participants who provided cervical swabs was 32.8 years (SD ± 9.0 years, range = 18–64 years).

The Xpert CT/NG test was performed on 1,764 specimens collected at three sites, with 91.0% (1,605/1,764) producing a valid result in the first test. There were 144 samples reported as indeterminant in the first run by the Xpert test, and four of them were also reported as indeterminant by the Roche test. There were 84 and 62 indeterminate results in the urine (12.8%, 84/657) and cervical samples (5.6%, 62/1,107), respectively. The majority (128/144, 89.9%) of the samples yielded valid results after retesting. Among these were 11 specimens reported after retesting as positive for CT and 5 specimens reported as positive for NG. Additionally, positive CT and NG results were reported for 20 and 10 specimens, respectively. All were initially reported as errors. Finally, among the specimens reported as “no result” in the first run, 11 were reported as positive CT, and 15 were reported as positive NG in the second run (**Table 1**). Among the 619 urine samples, 163 were CT positive and 121 were NG positive when using the Xpert assay, while 156 were CT positive and 125 were NG positive using the Roche test. Xpert reported 191 CT positive and 66 NG positive results among 1,042 cervical swabs. The Roche cobas^®^ 4800 CT/NG assay gave 177 CT positive and 60 NG positive results. There were 617 urine samples that reported the same CT results using the two tests and 11 urine samples that yielded different results between Xpert and Roche cobas (McNemar and Fisher’s exact test = 0.065). Similarly, there were 1,010 cervical swabs with the same results and 32 cervical samples with different results (McNemar’s *χ* ^2^ = 6.125, *p* = 0.013). Six hundred and thirteen urine samples gave concurrent NG results, but six urine samples reported different NG results by the Xpert and Roche cobas tests (McNemar and Fisher’s exact test = 0.219). A total of 1,036 cervical swabs had the same NG results, while six cervical samples (McNemar’s *χ* ^2^ = 6.000, *p* = 0.014) had different NG results detected by the Xpert and Roche tests (**Table 2**).

**Table 1:** Indeterminate results yielded in the first run and retested in the second run using the Xpert assay.

Indeterminate		Urine retest results		Cervical swabs retest results	
		CT	NG	CT	NG
Invalid	Negative	32	33	12	17
	Positive	2	1	9	4
ERROR	Negative	15	18	20	27
	Positive	10	7	10	3
No result	Negative	5	2	2	1
	Positive	11	14	0	1
Total		75	75	53	53

**Table 2:** Comparison of Cepheid GeneXpert^®^ and Roche cobas^®^ 4800 CT/NG tests for the detection of urogenital *Chlamydia trachomatis* (CT) and *Neisseria gonorrhoeae* (NG).

			Xpert-CT					Xpert-NG	
			Negative	Positive				Negative	Positive
Urine	Roche-CT	Negative	463	9	Roche-NG		Negative	493	1
		Positive	2	154			Positive	5	120
Cervical swab	Roche-CT	Negative	842	23	Roche-NG		Negative	976	6
		Positive	9	168			Positive	0	60

**Table 3** provides a summary of the concordance between the Xpert and Roche tests for the detection of urogenital CT and NG. For CT infection, the concordance was 98.2% (95%CI = 96.8–99.1) using the urine samples, with 98.6% (95%CI = 95.2–99.8) agreement among positive samples and 98.1% (95%CI = 96.4–99.1) agreement among negative samples. The concordance was 96.9% (95%CI = 95.7–97.9) for the cervical swabs, with 94.9% (95%CI = 90.6–97.7) agreement among positive samples and 97.3% (95%CI = 96.0–98.3) agreement among negative samples. For NG infection, the concordance was 99.0% (95%CI = 97.9–99.6) using the urine samples, with 96.0% (95%CI = 91.0–98.7) agreement among positive samples and 99.8% (95%CI = 98.9–100.0) agreement among negative samples. The concordance was 99.4% (95%CI = 98.8–99.8) for cervical swabs, with 100.0% (95%CI = 90.4–100.0) agreement among positive samples and 99.4% (95%CI = 98.7–99.8) agreement among negative samples.

**Table 3:** Concordance between Cepheid GeneXpert^®^ and Roche cobas^®^ 4800 CT/NG tests for detection of urogenital *Chlamydia trachomatis* (CT) and *Neisseria gonorrhoeae* (NG).

			Xpert-CT					Xpert-NG	
			Negative	Positive				Negative	Positive
Urine	Roche-CT	Negative	463	9	Roche-NG		Negative	493	1
		Positive	2	154			Positive	5	120
Cervical swab	Roche-CT	Negative	842	23	Roche-NG		Negative	976	6
		Positive	9	168			Positive	0	60

The discordant results were resolved by DNA sequence analysis, shown in **Table 4**. Three urine samples (27.3%) were confirmed by the DNA sequence analysis as having the same results as those by Xpert for the detection of CT among 11 samples with discordant results produced by the Xpert and Roche assays, and 19 sequence results for the cervical samples (59.4%, 19/32) were the same as those of Xpert for the detection of CT. Five urine samples (83.3%, 5/6) and three cervical swabs (50%, 3/6) showing discordant results were verified by DNA sequencing as having the same results as those by Xpert for the detection of NG (**Table 4**). For CT infection after discrepant resolution, the sensitivity and specificity of the Xpert assay using urine estimates were 100.0% (95%CI = 96.8–99.9) and 98.3% (95%CI = 96.6–99.2), respectively. The sensitivity and specificity using cervical swab estimates were 99.4% (95%CI = 96.5–100.0) and 98.6% (95%CI = 97.5–99.2), respectively. For NG infection, the sensitivity and specificity of the Xpert assay using urine samples were 99.2% (95%CI = 94.9–100.0) and 100.0% (95%CI = 99.0–100.0), respectively. The sensitivity and specificity using cervical swab estimates were 100% (95%CI = 92.8–100.0) and 99.7% (95%CI = 99.0–99.9), respectively (**Table 5**).

**Table 4:** Results of the validation for the discordant results detected by Cepheid GeneXpert^®^ (Xpert) and Roche cobas^®^ 4800 CT/NG tests for urogenital *Chlamydia trachomatis* (CT) and *Neisseria gonorrhoeae* (NG) .

Sequencing results		CT		Sequencing results		NG	
		Xpert positive and Roche negative	Xpert negative and Roche positive			Xpert positive and Roche negative	Xpert negative and Roche positive
CT-urine	Positive	1	0	NG-urine	Positive	1	1
	Negative	8	2		Negative	0	4
CT-swab	Positive	11	1	NG-swab	Positive	3	0
	Positive	12	8		Negative	3	0

**Table 5:** Sensitivity and specificity of the Cepheid GeneXpert^®^ CT/NG test for the detection of urogenital *Chlamydia trachomatis* (CT) and *Neisseria gonorrhoeae* (NG).

		Sensitivity (95%CI)	Specificity (95%CI)
Urine	CT	100.0% (96.8–99.9)	98.3% (96.6–99.2)
	NG	99.2% (94.9–100.0)	100.0% (99.0–100.0)
Cervical swabs	CT	99.4% (96.5–100.0)	98.6% (97.5–99.2)
	NG	100% (92.8–100.0)	99.7% (99.0–99.9)

## Discussion

This study is the first multicenter study in China to evaluate the performance of the Xpert CT/NG assay for the real-time simultaneous detection of chlamydia and gonorrhea using urine and cervical swab specimens. In this study, 8.3% of the specimens were initially reported either as “error”, “invalid”, or “no result” by the Xpert test, so the results could not be reported as either positive or negative. The reasons for indeterminate results include high sample viscosity, often due either to the presence of mucus, which interferes with amplification; or the presence of PCR inhibitors in the specimen, such as blood, which also gives an invalid result (Schrader et al., 2012; Parcell et al., 2015). After retesting, 89.9% of the samples with indeterminate results gave a valid result using the Xpert CT/NG test. This rate was comparable to that observed in a previous study with the cobas assay (i.e., ~80%) (Parcell et al., 2015). The results of the Xpert CT/NG test showed high concordance with the Roche cobas^®^ 4800 CT/NG results for the detection of CT and NG using urine specimens. There were no statistical differences between these two assays (McNemar and Fisher’s exact test = 0.065 and 0.219 for CT and NG, respectively), and the results demonstrated concordance for urine of 98.2% and 99.0% for CT and NG, respectively. The high concordance between the results of the Xpert and Roche cobas^®^ 4800 CT/NG assays for the detection of CT and NG has previously been reported for urine samples (Causer et al., 2018; Speers et al., 2018) and rectal swabs (Badman et al., 2019). The Xpert CT/NG test results also showed high concordance with those of Roche cobas 4800 CT/NG for the detection of CT and NG using cervical swabs (CT: 96.9%, 95%CI = 95.7–97.9; NG: 99.4%, 95%CI = 98.8–99.8). These two assays showed statistical differences (CT: McNemar’s *χ* ^2^ = 6.125, *p* = 0.013: NG: McNemar’s *χ* ^2^ = 6.000, *p* = 0.014). For CT infection, the sensitivity for both urine samples and cervical swabs was near perfect and the specificity values were 98.3% and 98.6% for urine samples and cervical swabs, respectively. For NG infection, the sensitivity and the specificity for the urine samples and cervical swabs were both higher than 99.0%. These results were comparable to those of previous evaluation studies (Gaydos et al., 2013; Herbst-de-Cortina et al., 2016; Bristow et al., 2019; Xie et al., 2020).

The Xpert CT/NG assay was the first nucleic acid-based test for CT and NG available for point-of-care (POC) use. Generally, this assay can be completed in 90 min, reducing the delay in reporting the results and potentially enabling rapid effective treatment. It satisfies the REASSURED criteria for the design of STI diagnostic tests: real-time connectivity, ease of specimen collection, affordable, sensitive, specific, user-friendly, rapid and robust, equipment-free or requiring only simple epuipment, environment-friendly, and deliverable to end-users (Land et al., 2019). Prior unpublished data suggested that more than half (225/446, 50.4%) of patients preferred to pay higher expenses to obtain highly effective detection (high sensitivity and specificity with a reduced turnaround time). Nearly 62.8% (279/446) of patients would prefer to pay less than 60 Yuan Renminbi (US $9.4) for CT infection tests. This means that, if the cost of this assay was less than US $20, nearly more than half of patients would choose this assay for a reduced reporting time with high performance.

There are some limitations to this study. Firstly,we did not evaluate the vaginal swabs for female patients. Vaginal swabs tend to be preferable among female patients due to reduced pain and easy collection. Secondly, this assay was performed by trained technicians in urban hospitals which may not reflect the performance of this assay in remote settings with clinicians in the real world.

The Xpert CT/NG assay exhibited high sensitivity and specificity for the detection of CT and NG in urine samples and cervical swabs. The short turnaround time of this assay can be useful in reducing the reporting time and enable more patients to receive treatments quickly, thus potentially improving chlamydia and gonorrhea control efforts. The one drawback to the assay is the initial number of indeterminate results, which was approximately 8%, although most were resolved on retesting.

## Data Availability Statement

The original contributions presented in the study are included in the article/supplementary material. Further inquiries can be directed to the corresponding author.

## Ethics Statement

The studies involving human participants were reviewed and approved by the Institute of Dermatology, the Chinese Academy of Medical Sciences and Peking Union Medical College, Dermatology Hospital of Southern Medical University, and The Third Affiliated Hospital of Guangzhou Medical University. The patients/participants provided written informed consent to participate in this study.

## Author Contributions

YH analyzed the data, interpreted the findings, and drafted the manuscript. MS and YY conceived the study and supervised all aspects of its implementation. QJ, HX, WL, and HZ recruited the subjects from the sites and examined the collected samples. WL recruited the subjects from the Nanjing site. MS examined the collected samples from the Nanjing site. FT and YT reviewed the drafts of the manuscripts. All authors contributed to the article and approved the submitted version.

## Funding

This study was funded by Danaher Diagnostic Platform China/Cepheid, Shanghai, China.

## Conflict of Interest

FT and YT are employees of Cepheid, the commercial manufacturer of the Xpert^®^ CT/NG test.

The remaining authors declare that the research was conducted in the absence of any commercial or financial relationships that could be construed as a potential conflict of interest.

## Publisher’s Note

All claims expressed in this article are solely those of the authors and do not necessarily represent those of their affiliated organizations, or those of the publisher, the editors and the reviewers. Any product that may be evaluated in this article, or claim that may be made by its manufacturer, is not guaranteed or endorsed by the publisher.
